# Expression of active trypsin-like serine peptidases in the midgut of sugar-feeding female *Anopheles aquasalis*

**DOI:** 10.1186/s13071-015-0908-0

**Published:** 2015-05-29

**Authors:** Geovane Dias-Lopes, Andre Borges-Veloso, Leonardo Saboia-Vahia, Gilberto B. Domont, Constança Britto, Patricia Cuervo, Jose Batista De Jesus

**Affiliations:** Laboratório de Biologia Molecular e Doenças Endêmicas – Instituto Oswaldo Cruz, FIOCRUZ, Rio de Janeiro, Brazil; Laboratório de Química de Proteínas, Departamento de Química, UFRJ, Rio de Janeiro, Brazil; Laboratório de Pesquisa em Leishmaniose – Instituto Oswaldo Cruz, FIOCRUZ, Av. Brasil 4365, Manguinhos, Pav. 26, Sala 509, Rio de Janeiro, Brazil; Departamento de Medicina, Faculdade de Medicina – Universidade Federal de São João Del Rey, São João del Rei, MG Brazil

**Keywords:** *Anopheles aquasalis*, Midgut, Zymography, Trypsin-like serine peptidases, Proteomics, Mass spectrometry

## Abstract

**Background:**

*Anopheles aquasalis* is a dipteran of the family Culicidae that is widely distributed in the coastal regions of South and Central America. This species acts as a vector of *Plasmodium vivax*, an important etiological agent of malaria, which represents a serious public health problem. In mosquitoes, trypsin-like serine proteases are important in blood meal digestion, immune responses and reproductive functions. The study of peptidases expressed in the mosquito midgut is essential to understanding the mechanisms of parasite-host interaction and the physiological process of nutrient digestion.

**Methods:**

Our study aimed to identify and characterize the proteolytic activities in the midgut of sugar-fed *An. aquasalis* females using zymographic analyses (substrate-SDS-PAGE), in-solution assays and mass spectrometry.

**Results:**

Here, we used a zymographic analysis to further biochemically characterize the proteolytic profile of the midgut of sugar-feeding *An. aquasalis* females. The trypsin peptidases migrated between ~17 and ~76 kDa and displayed higher proteolytic activities between pH 7.5 and 10 and at temperatures between 37 °C and 50 °C. Four putative trypsin-like serine peptidases were identified using mass spectrometry and data mining. The molecular masses of these peptidases were similar to those observed using zymography, which suggested that these peptidases could be responsible for some of the observed proteolytic bands.

**Conclusions:**

Taken together, our results contribute to the gene annotation of the unknown genome of this species, to the tissue location of these peptidases, and to the functional prediction of these crucial enzymes, which all impact further studies of this species.

## Background

*Anopheles (Nyssorhynchus) aquasalis* is a dipteran of the family Culicidae that is widely distributed in the coastal regions of South and Central America [1]. In these regions, this species acts as a vector of *Plasmodium vivax* [2, 3], an important etiological agent of malaria, which represents a serious public health problem [4–6]. The prevention of malaria transmission relies primarily on vector control strategies, which depend on the understanding of mosquito biology [7–10]. Understanding the physiology and expression of specific proteins in mosquito tissues that are involved in the pathogen-host interaction can provide relevant data for developing alternative measures to control the disease [11–16]. Currently, *An. aquasalis* is the only Neotropical anopheline for which an experimental autonomous colony can be established [17], which allows for their use as a controlled model for various studies.

Peptidases are proteolytic enzymes that can be subdivided into endopeptidases and exopeptidases [18]. Serine peptidases are a class of endopeptidases that are characterized by the presence of three invariant residues at the active site: serine, aspartic acid and histidine [19, 20]. In mosquitoes, serine peptidases are related primarily to food digestion, and blood feeding specifically regulates some of these enzymes in female adults [21–23]. In fact, this regulation is an important characteristic of hematophagic activity and is involved in host-pathogen interaction [24–26]. In *Aedes aegypti*, the midgut trypsin activity provides nutrients that sustain the dengue virus (DENV-2) replication and possibly act in viral surface processing, which enhances infection [27]. Experimental infections of *Ae. aegypti* with *Plasmodium gallinaceum* demonstrated that a mosquito’s trypsin-like peptidase activates the parasite’s prochitinase, which allows the *Plasmodium* to traverse through the peritrophic matrix [16]. In addition, serine peptidases are essential for mosquito immune responses, such as hemolymph coagulation, antimicrobial peptide synthesis, and the melanization of the pathogen [15, 27–29]. Furthermore, the serine peptidases of the female lower reproductive tissues of *An. gambiae* are involved in the processing of male products transferred to females during mating [30].

Despite the availability of data on the role of serine peptidases in the mosquitoes’ blood digestion, few studies have examined the active peptidases expressed in the midgut of anophelines females that feed on sugar [31–33]. Our study aimed to identify and characterize the proteolytic activities in the midgut of sugar-fed *An. aquasalis* females using zymographic analyses (substrate-SDS-PAGE), in-solution assays and mass spectrometry. We demonstrated that sugar-feeding *An. aquasalis* females exhibit a complex profile of trypsin-like serine peptidases with high activity in alkaline pH, and we identified four trypsin genes that are expressed at the protein level in the midgut of the females. Given that the expression at the protein level of peptidase genes and the demonstration that they are active in female mosquitoes fed on sugar are controversial issues, our results contribute to clarifying such subjects as we characterized the active peptidases profile and identified trypsin-like serine peptidases by mass spectrometry. The role of the enzymes expressed under such conditions should be clarified in future works.

## Methods

### Chemicals

Stock solutions of 1,10-phenantroline (200 mM) and pepstatin A (1 mg/ml) were prepared in ethanol, whereas trans-epoxysuccinyl L-leucylamido-(4-guanidino) butane (E-64, 1 mM) was prepared in water. Phenyl-methyl sulfonyl-fluoride (PMSF, 250 mM) was diluted in isopropanol, and N-a-Tosyl-L-lysine chloromethyl ketone hydrochloride (TLCK, 100 mM) and N-p-Tosyl-L-phenylalanine chloromethyl ketone (TPCK, 100 mM) were prepared in methanol. All protease inhibitors were maintained at - 20 °C.

### Insects

The experiments were conducted with female *An. aquasalis* adults that were obtained from a colony established and maintained in the Laboratório de Fisiologia e Controle de Artrópodes Vetores of the Instituto Oswaldo Cruz (Rio de Janeiro). The mosquitoes were fed a 10 % sugar solution *ad libitum* and maintained in laboratory conditions at 26–28 °C and 70–80 % relative humidity. For each experiment, the female adults (3–5 days old) were cold-anesthetized on ice and decapitated. The midgut was dissected as previously described [34].

### Zymographic analysis

Pools of 20 midguts were washed twice with phosphate-buffered saline (PBS), pH 7.2, and homogenized in lysis buffer containing 10 % glycerol, 0.6 % Triton X-100, 100 mM Tris–HCl pH 6.8, and 150 mM NaCl [35]. The homogenates were centrifuged at 14,000 × *g* and 4 °C for 30 min to remove insoluble material, and the proteins were resolved as previously described [36]. Briefly, the supernatants were mixed with sodium dodecyl sulfate polyacrylamide gel electrophoresis (SDS-PAGE) sample buffer (125 mM Tris, pH 6.8, 4 % SDS, 20 % glycerol, 0.002 % bromophenol blue) and loaded onto 10 % SDS-PAGE co-polymerized with 0.1 % porcine gelatin. The gels were loaded with 5 μg of protein per well, and electrophoresis was carried out at 4 °C with a constant voltage of 110 V. Subsequently, the gels were washed twice for 30 min at 4 °C in either 100 mM sodium acetate buffer (pH 3.5 or 5.5) containing 2.5 % Triton X-100 or 100 mM Tris–HCl buffer (pH 7.5 or 10.0) plus 2.5 % Triton X-100. The protease activities were detected by incubating the gels in a reaction buffer containing 100 mM sodium acetate (pH 3.5 or 5.5) or 100 mM Tris–HCl buffer (pH 7.5 or 10.0) at 37 °C for 30 min, 2, 4, and 6 h. The hydrolyzed gelatin bands were visualized by staining the gels in a solution of 0.2 % Coomassie blue R-250, 40 % methanol, and 10 % acetic acid and de-staining them with 10 % acetic acid. The molecular masses of the peptidases were estimated by comparing them with the mobility of molecular mass standards (PageRuler™ Protein Ladder, Fermentas). All results are derived from three independent experiments carried out in triplicate.

### Effect of temperature and peptidase inhibitors on proteolytic activity

After the electrophoresis, gels of female midgut homogenates were incubated at 10, 25, 37, 50, 65 and 80 °C for 2 h in preheated 100 mM Tris–HCl pH 7.5 reaction buffer. The peptidases were resolved as described above. To analyze the sensitivity of peptidases to inhibitors, the female midgut homogenates were pre-incubated (before electrophoresis) for 30 min at 37 °C with one of the following peptidase inhibitors: 10 μM E-64, 1 mM PMSF, 100 μM TLCK, 100 μM TPCK, 10 μM pepstatin-A, or 10 mM 1,10-phenanthroline. These inhibitors were also added to the reaction buffer at the same concentration. The peptidase activities were resolved as described above.

### In-solution enzymatic assays

The in-solution assays were performed at 37 °C and pH 7.5 using the fluorogenic substrate Z-carbobenzoxy-L-phenylalanyl-L-arginine-(7-amino-4-methylcoumarin) [Z-Phe-Arg-AMC]. The substrate was prepared at a concentration of 3 mM in dimethyl sulfoxide (DMSO) and diluted to a 100 μM working concentration for each assay. The reactions proceeded by adding 5 μg of proteins from the female midgut diluted in 100 mM sodium acetate (at pH 3.5 or 5.5) or 100 mM Tris–HCl (pH 7.5 or 10.0) for pH evaluation or peptidase inhibitors: 10 μM E-64, 1 mM PMSF, 100 μM TLCK, 100 μM TPCK. The fluorescence intensity was measured continuously by spectrophotofluorometry for a 45 min period (Molecular Devices) using excitation and emission wavelengths of 380 and 460 nm, respectively. All assays were performed at 37 °C. Controls without enzyme or without substrate were also included. All results are derived from three independent experiments carried out in triplicate.

### Protein extraction and mass spectrometry analysis

Fifty pooled midguts were mechanically disrupted with a pestle and a motor drive in 100 μL of lysis buffer (2 M Thiourea, 7 M Urea, 4 % CHAPS and 65 mM DTT) and a protease inhibitor cocktail. To remove the insoluble material, the samples were centrifuged at 14,000 × *g* for 15 min at 4 °C. Subsequently, the samples were precipitated with a 3:1 solution of methanol:chloroform. The proteins were re-suspended in buffer containing 0.5 M Hepes and 7 M urea, and the protein concentration was determined using Qubit. The proteins were enzymatically digested in a solution following previously established protocols [37] with some modifications. Briefly, the proteins were reduced in 65 mM DTT for 30 min at 56 °C and then alkylated with 200 mM iodoacetamide at 25 °C in the dark for 30 min. Trypsin (200 ng) was added, and the mixture was incubated at 37 °C overnight. The peptides were purified and concentrated using a column packed in-house with Poros oligo R3 resin (Applied Biosystems). The peptides were finally eluted with a solution of 0.1 % formic acid in 50 % v/v acetronitrile. Three biological replicates were performed in technical triplicates.

For each sample, 4 μL of peptides were applied to an EASY II-nano LC system (Thermo Fisher Scientific) coupled online to an ESI-LTQ-Orbitrap Velos mass spectrometer (Thermo Fisher Scientific). The peptides were eluted through a trap column (150 μm × 2 cm) packed in-house with C-18 ReproSil 5 μm resin and an analytical column (100 μm × 15 cm) packed in-house with C-18 ReproSil 3 μm resin. Mobile phase A consisted of 0.1 % (v/v) formic acid in water, and mobile phase B consisted of 0.1 % (v/v) formic acid in acetonitrile. The gradient conditions were as follows: 5–40 % B in 180 min. Mass spectra were acquired in positive mode using the data-dependent automatic (DDA) survey MS scan and tandem mass spectra (MS/MS) acquisition. Each DDA consisted of a survey scan of the m/z range of 300–2000 and resolution of 60 000 with a target value of 1 × 10^−6^ ions. Survey scan was followed by the MS/MS of the 10 most intense ions in the LTQ using the collision-induced dissociation (CID), and previously fragmented ions were dynamically excluded for 60 s.

### Protein identification

The data were searched using the ProLuCID 1.3 search engine in the PatternLab platform [38] against a customized database that included non-redundant sequences of the Culicidae family retrieved from UniRef100 (approximately 81,000 entries, downloaded June 2014, http://www.uniprot.org/). Searches were performed with one missed cleavage, with the carbamidomethylation of cysteine residues as a fixed modification, methionine oxidation as a variable modification and mass tolerances of 50 ppm and 500 ppm as precursor and fragment ions, respectively. The false discovery rate (FDR) was estimated using the identifications obtained with the reversed decoy database. The validity of the peptide sequence matches (PSMs) was assessed using the Search Engine Processor (SEPro) of the PatternLab platform [39]. This software uses the XCorr, DeltaCN and ZScore values from the ProLuCID analysis to create a discriminator score. The peptide identification was based on a false-discovery rate (FDR) of 1 % and post-processed to only accept PSMs mass tolerances less than 10 ppm. The protein identification is supported by at least two independent pieces of evidence: (i) the identification of a peptide with different charge states; (ii) a modified and a non-modified version of the same peptide; (iii) more than two spectra for peptide; or (iv) two different peptides.

### Multiple sequence alignment analysis

The amino acid sequences of trypsin peptidases identified by MS/MS were aligned using CLUSTAL Omega [40] against the well-annotated sequences of *Ae. aegypti* trypsin 3 (TRY3_AEDAE) and *An. gambie* trypsin 6 (TRY6_ANOGA). The trypsin sequences were scanned for the active site (His, Asp, Ser), the signal peptide and the conserved cysteine residues of disulfide bounds using the PROSCAN function of the PROSITE suite (http://prosite.expasy.org) [41]. The signal peptide was also predicted by SignalP 4.0 (http://cbs.dtu.dk/services/SignalP) [42].

## Results

### Time course of proteolytic activities in the midgut of *An. aquasalis* females

**Fig. 1:**
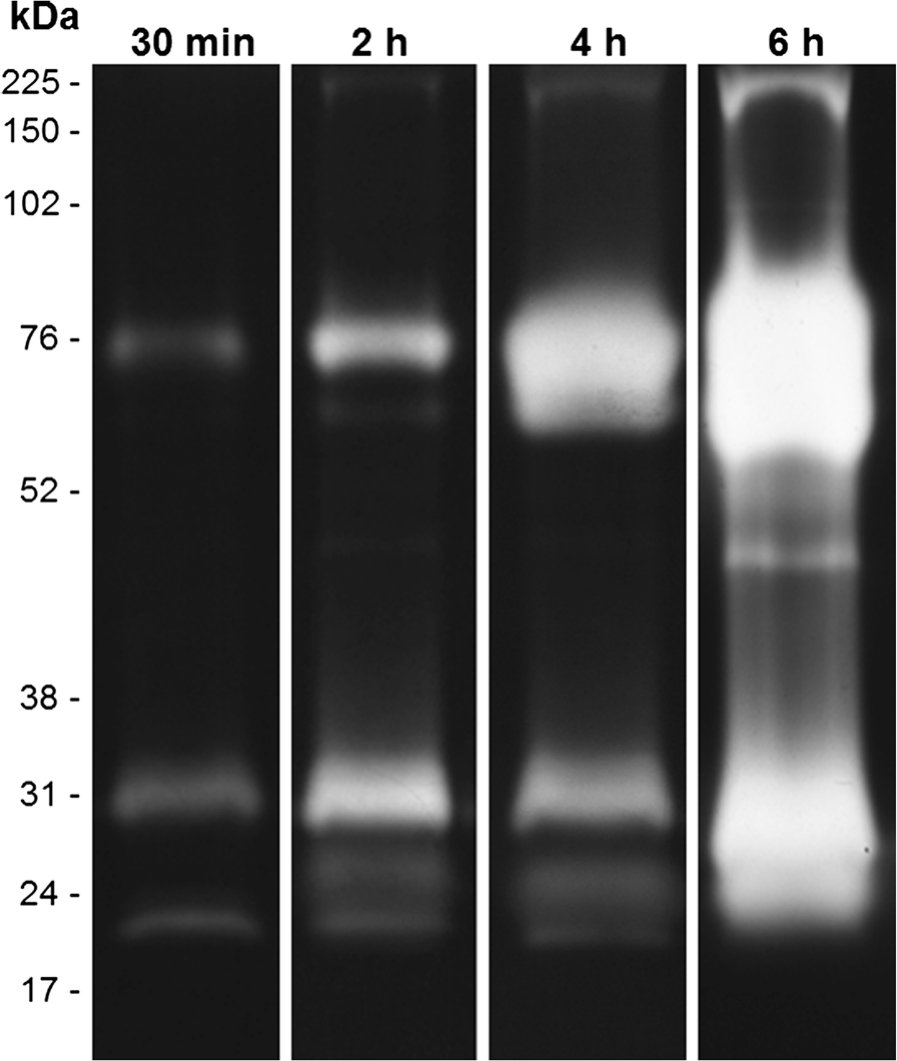
Time-course of the proteolytic activities exhibited by the midgut of *An. aquasalis* female. Proteolytic activities were detected after incubating the gels at 37 °C in 0.1 M Tris–HCl buffer (pH 7.5) for 30 min, 2, 4 and 6 h. The numbers on the left indicate the apparent molecular masses of the active bands expressed in kDa

To evaluate the influence of the reaction time on the proteolytic activity of the midgut from *An. aquasalis* females, the gels were incubated for 30 min, 2, 4 and 6 h in 100 mM Tris–HCl reaction buffer (pH 7.5) at 37 °C (Fig. 1). The intensity of the proteolytic activity progressively increased up to 6 h. After 2 h of reaction, the proteolytic profile consisted of at least seven bands ranging from ~17 to ~76 kDa. At 4 and 6 h, the intense enzymatic activity led to the overlapping of the bands, which precluded the analysis. Thus, the enzymatic reaction time was limited to 2 h for the subsequent analyses.

### Effect of pH on the proteolytic profile of the midgut of *An. aquasalis* females

**Fig. 2:**
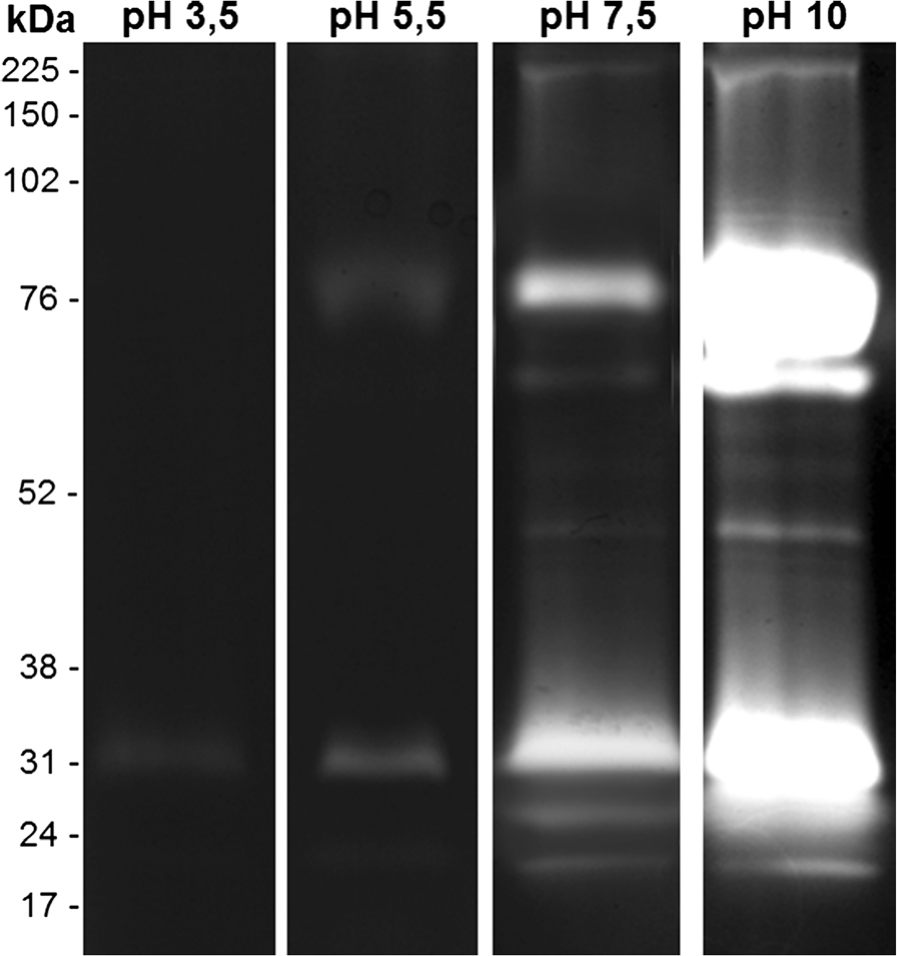
Effect of pH on the proteolytic profiles of the midgut of *An. aquasalis* female. Enzymatic activities were evaluated after incubating the gels for 2 h at 37 °C in reaction buffer containing 100 mM sodium acetate at pH 3.5 or 5.5 or 100 mM Tris–HCl at pH 7.5 or 10.0. The numbers on the left indicate the apparent molecular masses of the active bands expressed in kDa

To determine the effect of the pH on the proteolytic activity, the gels were incubated for 2 h in reaction buffer at pH 3.5, 5.5, 7.5 or 10. The enzymatic activities were detected at all tested pH values and progressively increased from pH 3.5 to 10 (Fig. 2). At pH 3.5 and 5.5, only a few bands were detected. These bands were less intense than the bands obtained at pH 7.5 and 10. At pH 10, the intense proteolytic activity hampered the resolution of the profile. Thus, the proteolytic profile was better resolved at pH 7.5.

### Influence of temperature and peptidase inhibitors on the proteolytic profile of the midgut of *An. aquasalis* females

**Fig. 3:**
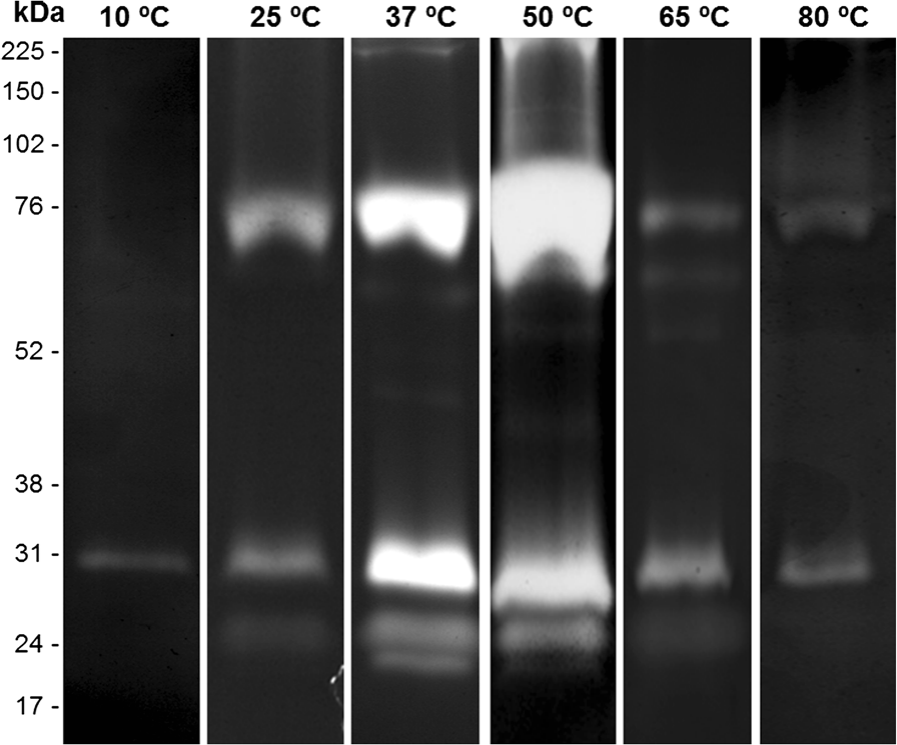
Influence of temperature on the proteolytic profiles of the midgut of *An. aquasalis* female. Enzymatic activities were detected after incubating the gels for 2 h at 10, 25, 37, 50, 65, or 80 °C in reaction buffer containing 100 mM Tris–HCl at pH 7.5. The numbers on the left indicate the apparent molecular masses of the active bands expressed in kDa

**Fig. 4:**
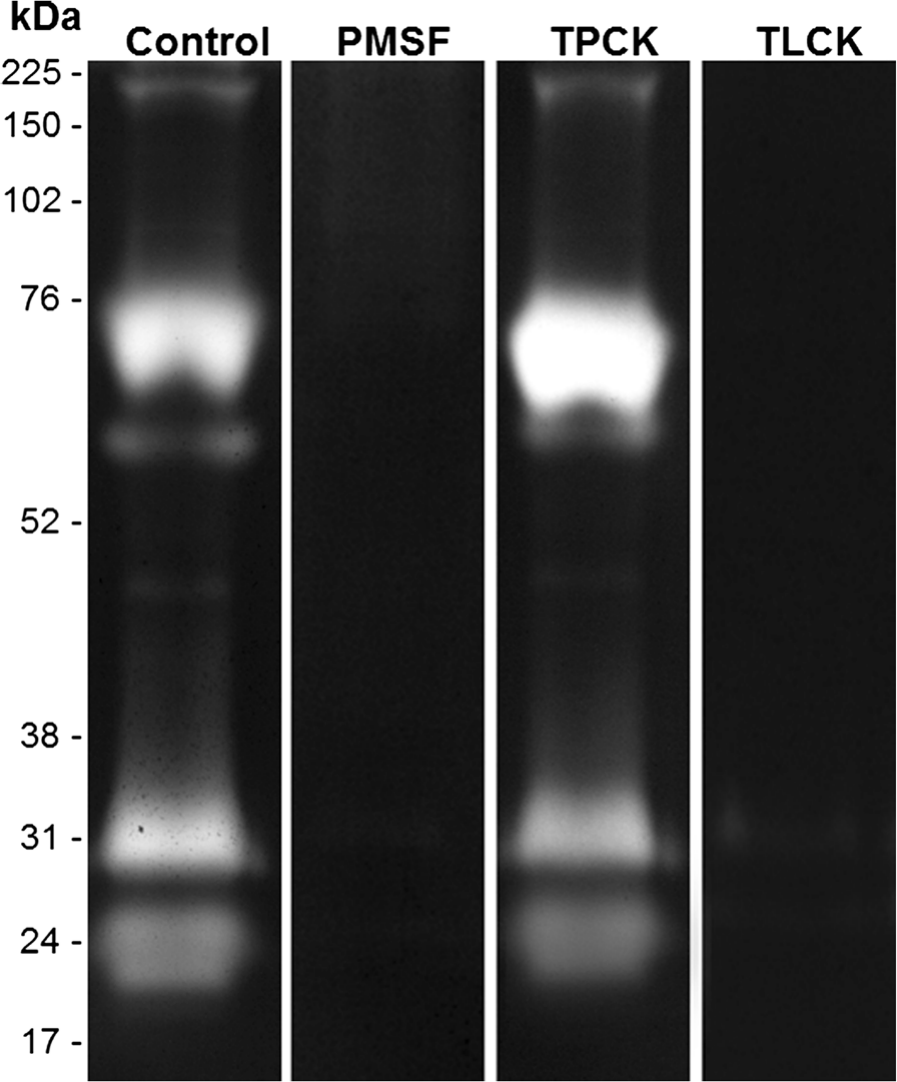
Effect of protease inhibitors on the proteolytic profiles of the midgut of *An. aquasalis* females. Peptidase activities were detected after incubating the gels for 2 h at 37 °C in 100 mM Tris–HCl pH 7.5. Proteolytic assays were performed in the absence (control) or presence of each of the following protease inhibitors: 1 mM PMSF; 100 μM TLCK; or 100 μM TPCK. The numbers on the left indicate the apparent molecular masses of the active bands expressed in kDa

The thermal sensitivity of the enzymatic activity of female midguts was evaluated by incubating the gels for 2 h at 10, 25, 37, 50, 65 or 80 °C in 100 mM Tris–HCl reaction buffer at pH 7.5 (Fig. 3). The enzymatic activity at 10 and 25 °C was low compared with that observed at 37 °C. The activity strongly increased at 50 °C and then decreased at 65 °C and 80 °C. The proteolytic profile exhibited by the midgut of females was strongly inhibited by 1 mM PMSF and 100 μM TLCK. However, 100 mM TPCK did not affect the proteolytic activity (Fig. 4). The activities were not inhibited by 10 mM E-64, 10 mM pepstatin A, or 10 mM 1,10-phenanthroline (data not shown). These results demonstrate that the trypsin-like serine peptidases are responsible for the observed activity.

### In-solution enzymatic assays

**Fig. 5:**
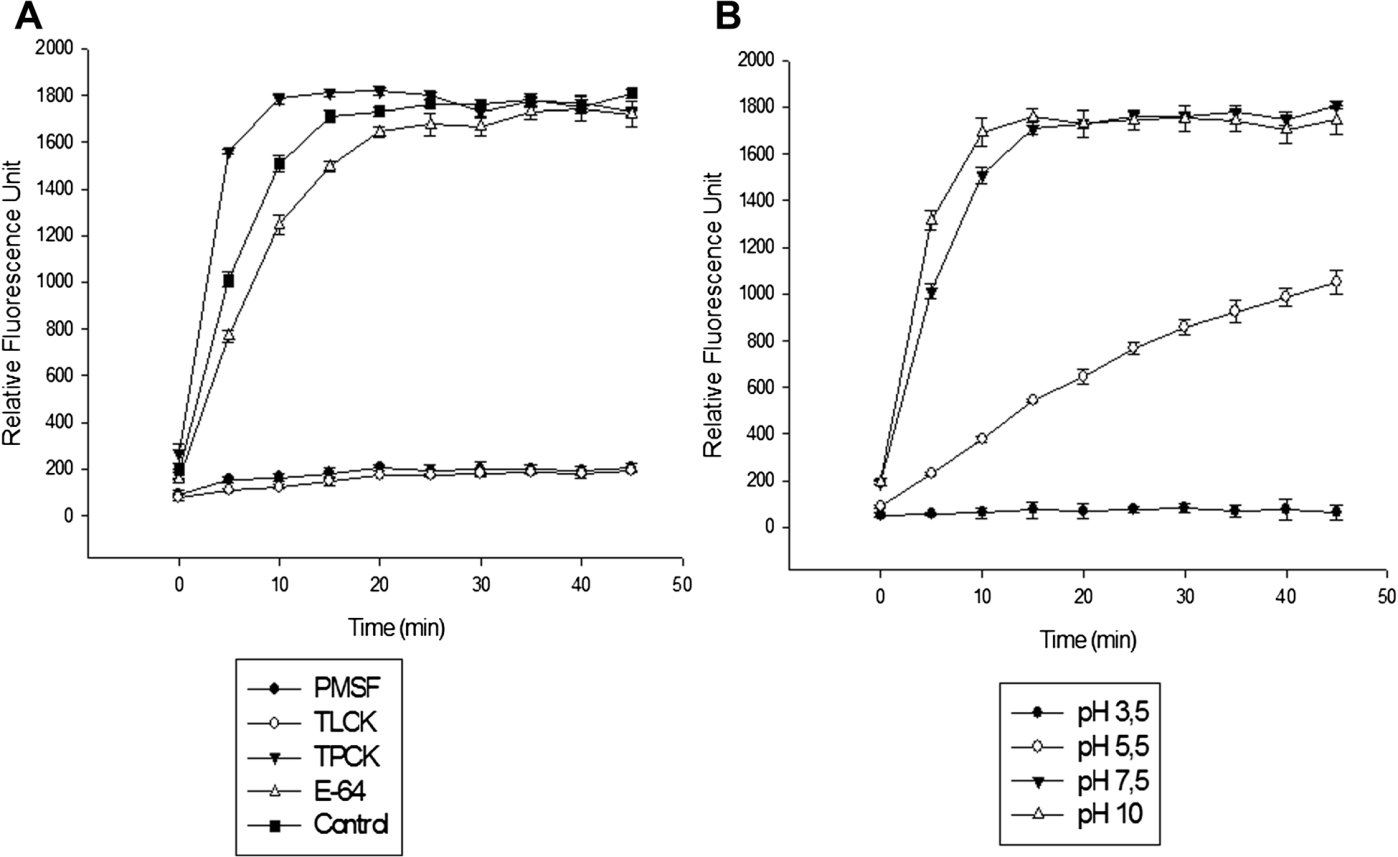
Detection of proteolytic activity from the midgut of *An. aquasalis* females using the fluorogenic substrate Z-Phe-Arg-AMC. **a** The proteolytic activity was detected using the substrate in the absence (control) or presence of 1 mM PMSF, 100 μM TLCK, 100 μM TPCK, 10 μM E-64 in 100 mM Tris–HCl buffer, pH 7.5. **b** Effect of pH on the proteolytic activity. Assays were performed at 37 °C in a 100 mM sodium acetate (pH 3.5 and 5.5) buffer or 100 mM Tris–HCl buffer (pH 7.5 and 10)

The effects of the peptidase inhibitors and pH on the proteolytic activities of midgut homogenates were also evaluated by in-solution assays using the fluorogenic substrate Z-Phe-Arg-AMC (Fig. 5). PMSF and TLCK, but not TPCK, strongly inhibited these activities, which corroborated the in-gel results (Fig. 5a). In addition, enzymatic activity was detected at all of the tested pH values, and this activity progressively increased from pH 3.5 to 10. At pH 7.5 and 10, the hydrolysis of the substrate reached a plateau after 15 min of reaction and remained stable until the final reaction time of 45 min (Fig. 5b).

### Identification of trypsin-like serine peptidases using mass spectrometry

**Fig. 6:**
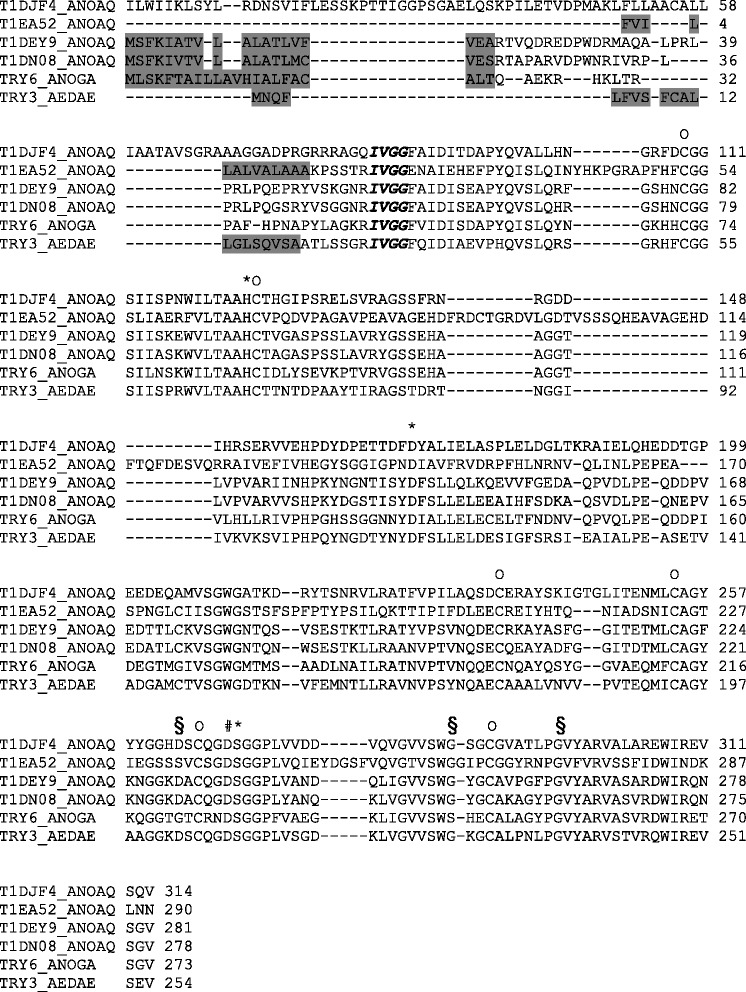
Alignment of *An. aquasalis* trypsin sequences identified by MS/MS with well annotated trypsin sequences (trypsin-3 of *Ae. aegypti* and trypsin-6 of *An. gambiae*). Regions of importance are represented as follows: (Gray) signal peptide; (Italic and bold) N-terminal residues of the active enzyme; (O) conserved cysteine of disulfide bonds; (*) conserved catalytic triad; (§) accessory catalytic residues; (#) highly conserved Asp 194

Four trypsin peptidases were identified in the midgut of sugar-fed females of *An. aquasalis* using mass spectrometry (Table 1). These peptidases were identified by matching them to the *An. aquasalis* sequences in the UniRef100 database. The molecular masses of the peptidases identified by MS ranged from 29 to 33 kDa. In addition, the peptide sequences identified by MS/MS for each peptidase were different. All identified trypsin proteins matched the *An. aquasalis* protein sequences. An analysis of the alignment of the amino acid sequence of these peptidases showed the following: (i) the conserved amino acid residues of the catalytic triad (His, Asp and Ser); (ii) the six cysteine residues involved in the disulfide bonds; (iii) the signal peptide sequence; and (iv) the putative autocatalytic activation motifs immediately after an arginine or lysine residue (R/K- IVGG) (Fig. 6).

**Table 1:** *Anopheles aquasalis* midgut trypsins identified using the ProLuCID and SEPro software

ID	Peptides	Length	MolWt (MH)	SeqCount	SpecCount	Coverage	Description
T1DJF4		314	33666,9	11	175	0,27	Putative trypsin-like serine protease Anopheles aquasalis
	R.EWIREVSQV.-						
	R.VALAREWIREVSQV.-						
	R.ATFVPILAQSDCER.A						
	R.AGSSFRNRGDDIHRSERVVEHPDYDPETTDFDYALIELASPLELDGLTKR.A						
	R.VVEHPDYDPETTDFDYALIELASPLELDGLTKR.A						
	R.VLRATFVPILAQSDCER.A						
	R.VALAREWIR.E						
	R.AGSSFRNRGDDIHR.S						
	R.ATFVPILAQSDCERAYSK.I						
	R.VLRATFVPILAQSDCERAYSK.I						
T1EA52		290	31253,4	1	23	0,05	Putative serine protease Anopheles aquasalis
	R.VSSFIDWINDKLNN.-						
T1DEY9		281	30124	8	99	0,37	Putative trypsin-3 Anopheles aquasalis
	R.DWIRQNSGV.-						
	R.YGSSEHAAGGTLVPVAR.I						
	D.PVEDTTLCKVSGWGNTQSVSESTKTLR.A						
	R.VASARDWIRQNSGV.-						
	R.ATYVPSVNQDECR.K						
	R.FGSHNCGGSIISKEWVLTAAHCTVGASPSSLAVRYGSSEHAAGGTLVPVAR.I						
	K.TLRATYVPSVNQDECR.K						
	R.FGSHNCGGSIISKEWVLTAAHCTVGASPSSLAVR.Y						
T1DN08		278	29534,6	5	117	0,14	Putative trypsin-1 Anopheles aquasalis
	R.VASVRDWIRQNSGV.-						
	R.DWIRQNSGV.-						
	R.YGSSEHAAGGTLVPVAR.V						
	K.AGYPGVYAR.V						
	R.VASVRDWIR.Q						
	R.VASVRDWIR.E

## Discussion

Information on the serine peptidase expression in *An. aquasalis* is scarce, and only two studies have described the identification of two blood-induced trypsins (AnaqTryp-1 and AnaqTryp-2) [43] and two chymotrypsins (Anachy1 and Anachy2), which were detected 24 h after the ingestion of blood [44]. In addition, the characterization of one transcript that codes for a chymotrypsin-like peptidase of *An. aquasalis* expressed after infection with *P. vivax* has been reported [45]. Differences in the expression of distinct serine peptidase-coding transcripts during the different developmental stages of *An. aquasalis* and between sugar- and blood-feeding females have been described [46]. In this work, we demonstrated the activity of trypsin-like serine peptidases in the midgut of sugar-feeding females and identified, by MS/MS, peptidases with molecular masses similar to those observed in the zymography, which suggested that these peptidases could be responsible for some of the observed proteolytic bands.

The epithelial cells of the midgut of anopheline mosquitoes release apical vesicles into the midgut lumen upon blood feeding. These vesicles contain trypsins and peritrophic matrix proteins, such as perithrofin 1, which are crucial for food digestion [47]. Because the stretching of the gut is the main stimulus for the release of these vesicles, even the intake of sugar promotes their release [32]. If trypsin peptidases released from vesicles find their substrate, the small peptides or free amino acids produced are the signal responsible for the transcription and translation of other genes related to digestion [32]. In fact, the trypsin genes of *An. gambiae* and *An. stephensi* contain putative trypsin regulatory elements (PTRE) that can bind to two distinct nuclear protein complexes responsible for the transcriptional regulation of early and late trypsins [48]. In *Ae. aegypti*, the early trypsin gene is transcribed before the blood meal, but its translation depends on the intake of blood [49]. The digestion of saline or sugar solutions cannot stimulate the translation of early trypsin; only the intake of amino acids is a sufficient stimulus. Amino acids have been demonstrated to be capable of inducing gene translation via the activation of the TOR kinase pathway [50]. Our study identified four trypsin-like serine peptidases and one peritrophin (Putative peritrophin-1 – data not shown) by MS/MS, which supported the expression of such proteins in females that fed on sugar.

An analysis of the transcriptome of sugar-fed *An. aquasalis* females revealed the expression of trypsin-like peptidases. These enzymes have been suggested to be involved in the initial steps of blood digestion or could be related to the regulation of other trypsin genes [46]. Similarly, early works showed that *An. gambiae* expresses a cluster of seven trypsin genes, among which antryp1 and antryp2 are induced after blood feeding, while antryp3, antryp4 and antryp7 are constitutively expressed and undetectable after blood feeding. The antryp1 and antryp4 zymogens are present in the apical vesicles of female midgut prior to a blood meal [33, 51]. These zymogens are activated during their secretion into the midgut lumen [47], likely via an auto-activation mechanism [33]. In agreement with these observations, we detected a high proteolytic activity in a short reaction time in *An. aquasalis* females that fed on sugar. In addition, our results show a drastic difference in the trypsin activity profile among Culicidae members fed on sugar [52]. These findings could suggest the existence of distinct mechanisms of trypsin regulation in such mosquito species. Together with fluid reduction via diuresis, which is mainly responsible for the mosquito body weight reduction after blood feeding, the high level of trypsins continuously expressed in the midgut of anopheline females may allow the mosquitoes to immediately digest the protein input, contributing also for the rapid reduction in body weight that would be important for females quick dispersion and oviposition as well as for host-seeking [31].

The molecular weight of trypsins found in the midgut of mosquitoes ranges from 20 to 35 kDa [23]. In agreement with this description, the masses of the four trypsin peptidases identified here by mass spectrometry ranged from 29 to 33 kDa. However, the zymographic assays indicated bands migrating between ~17 to ~76 kDa. The higher molecular mass bands could be due to the sample preparation procedure, in which the proteins are not completely denatured and reduced, enabling protein aggregation and/or oligomerization that slows the electrophoretic migration; therefore, the apparent molecular mass of peptidases may increase in the zymographic gel. Despite this possibility, the zymographic profiles observed here are highly reproducible and agree with previous reports of high molecular mass peptidase activities detected by zymographic methods [26, 52–56].

The pH of the midgut lumen is alkaline and can directly modulate the enzymatic activity [23]. The maintenance of the pH is attributed to the presence of proton pumps in the intestinal epithelium [57] and the enzyme carbonic anhydrase [58]. In the posterior midgut, where the blood is stored, peritrophic membrane proteins and digestive enzymes are secreted for the digestive process [59]. The pH of the posterior midgut of *Ae. aegypti* adult females ranges from 8.5 to 9.5, whereas it ranges from 8.0 to 9.5 in *An. gambiae* [58]. In agreement with these observations, our zymographic results showed that the proteolytic activity is higher at pH 7.5 and 10. These findings were also verified during the in-solution assays.

The serine peptidases of *Ae. aegypti* [55], *Oestrus ovis* [60] and the coleoptera *Tenebrio molitor* larvae [61, 62] are reportedly thermally sensitive. We observed that the trypsin-like serine peptidases of *An. aquasalis* are active over a large temperature range, but the proteolytic activity is markedly decreased at lower temperatures. These findings are in agreement with that observed in *O. ovis* [60]. The authors of this previous study suggested that these enzymes could be partially inactivated at low temperatures, which could affect the degradation of food in the larval stages and culminate in the low rates of larval development that are characteristic of the cold months in temperate climates [60]. The possibility that these enzymes retain some level of activity at low temperatures may have ecological implications in mosquitoes, such as during diapause, as suggested by other authors [63].

## Conclusion

The study of peptidases expressed in the mosquito midgut is essential to understand the mechanisms of parasite-host interaction and the physiological process of nutrient digestion. Here, we biochemically characterized the active proteolytic profile of *An. aquasalis* and confirmed the expression of four putative trypsin-like serine peptidases of similar molecular mass as those observed with zymography. These findings contribute to the gene annotation of the unknown genome of this species, to the tissue location of these peptidases, and to the prediction of the function of these crucial enzymes, impacting further studies of this species.
